# Simulation of citrus foliar gas exchange across diverse meteorological conditions: application of the optimal stomatal regulation method

**DOI:** 10.3389/fpls.2026.1748139

**Published:** 2026-03-12

**Authors:** Mengying Fan, Zhihui Wang, Xuelian Jiang, Ningbo Cui, Jingtian Zhao, Shouzheng Jiang, Guoyu Zhu, Liwen Xing, Xiaoxian Zhang

**Affiliations:** 1 State Key Laboratory of Hydraulics and Mountain River Engineering and College of Water Resource and Hydropower, Sichuan University, Chengdu, Sichuan, China; 2 Key Laboratory of Agricultural Planting Quantization and Application, Weifang University, Weifang, Shandong, China; 3 School of Computer Science and Technology, Algoma University, Sault Ste.Marie, ON, Canada; 4 Sustainable Crops and Soils, Rothamsted Research, Harpenden, United Kingdom

**Keywords:** citrus, meteorological factors, photosynthetic limitations, stomatal conductance, the optimal stomatal regulation

## Abstract

**Introduction:**

The optimal stomatal regulation theory provides an eco-evolutionary framework for interpreting the trade-off between CO_2_ uptake and water loss. This theory postulates that the marginal water cost of carbon gain (*λ=∂E/∂A*) remains approximately constant over short timescales, thereby offering a mechanistic basis for predicting stomatal behavior and gas exchange.

**Methods:**

In this study, leaf-level meteorological variables and gas exchange parameters of orchard citrus were measured throughout the entire phenological period during 2021–2022. We developed a family of optimal stomatal conductance-based models (OSCMs), comprising six forms: Rubisco-limited forms (OSCvc and OSCvcd), RuBP-regeneration-limited forms (OSCvj and OSCvjd), and combined forms that dynamically select the prevailing biochemical limitation (OSC and OSCd).

**Results:**

The key parameter *λ* was estimated daily and averaged over the entire phenological period. Using daily *λ* inputs, the three models produced stomatal conductance (*g_s_*) with accuracies ranked as OSCvjd (R^2^ = 0.73) > OSCd (0.63) > OSCvcd (0.40). When a long-term constant *λ* was applied, model performance declined with accuracies ranked as OSCvj (0.66) > OSC (0.52) > OSCvc (0.38).

**Discussion:**

The OSC model also produced intercellular CO_2_ concentration (*c_i_*) and photosynthesis (*A*) reasonably well (R^2^ = 0.78 and 0.48, respectively). Under moderate meteorological conditions (air temperature 30–40 °C and vapor pressure deficit 1–2 kPa), the OSC model showed its best performance with a mean absolute relative error of 35.2% for *g_s_* estimation. Overall, the OSCMs provided a mechanistic approach to simulate citrus leaf gas exchange requiring minimal species-specific traits and routine meteorological inputs. This modeling strategy supports rapid assessment of plant physiological status and estimation of foliar carbon-water fluxes in orchard management under subtropical climates.

## 1 Introduction

Citrus, characterized by millennia-long cultivation history, extensive varietal diversity, distinctive flavors, and high nutritional value, plays an essential role in human diet and agro-processing industry (Palangasinghe et al., 2024). As for 2023, the global citrus planting area has exceeded 8.76×10^6^ hectares and the annual production has reached 1.35×10^8^ tons (FAOSTAT, 2025). China contributes nearly 30% of global citrus production, making citrus cultivation a critical component of its agricultural economy, particularly in the hilly regions of Southwest China (Wang et al., 2022). However, regional water scarcity and frequent seasonal droughts have impeded sustainable citrus production (Dong et al., 2024). Such water-related constraints highlight the importance of stomatal regulation in coordinating water loss and carbon assimilation.

Stomata, the primary low-resistance gateway for leaf-atmosphere gas exchange, affect photosynthesis and transpiration directly (Negi et al., 2014). Stomatal function is especially dominant in citrus, where thick cuticles substantially restrict gas diffusion through epidermis (Tominaga and Kawamitsu, 2024). Stomatal aperture can rapidly respond to biotic and abiotic changes, while stomatal anatomy and density adapt more slowly to local climates over evolutionary time (Hetherington and Woodward, 2003). Field studies illustrate pronounced spatiotemporal variability in citrus stomatal behavior: well-managed *Citrus sinensis* L. in South Africa showed seasonal variation with peak stomatal conductance (*g_s_*) of 0.14 mol·m^-2^·s^-1^ in warm Autumn (Munjonji et al., 2021). In southeastern Brazil, an increase in atmospheric vapor pressure deficit (*D*) from 1.0 to 3.0 kPa reduced *g_s_* by 51% (Souza et al., 2004). On Corsica Island in southern France, the *g_s_* of *Citrus deliciosa* Ten. and *Citrus maxima* Merr. declined by 33–34% during the cold period, whereas the reduction reached 67% in *Citrus medica* L (Santini et al., 2012). Although many studies have investigated citrus stomatal responses, observed patterns differ across regions, climates, and cultivars, highlighting the complexity of stomatal regulation. Therefore, a unified theoretical framework is needed to interpret and predict stomatal behavior across diverse environments.

Researchers have proposed multiple explanations for stomatal behavior. Some argue that stomata respond directly to environmental factors (Jarvis and P., 1976), whereas others suggested that stomatal movement covaries with photosynthesis under given conditions (Ball et al., 1987), and still others emphasized the regulatory role of internal leaf states such as water potential, ion fluxes and cell turgor (Buckley et al., 2003; Delwiche and Cooke, 1977). Amid these diverse perspective, Cowan and Farquhar (1977) proposed a theory of optimal stomatal regulation from an evolutionary and philosophical interpretative angle: an acclimated plant would maximize its carbon gain over a finite water supply, mathematically formulated as maximizing the time integral of *A-E*/*λ*. Here, parameter *λ*—the marginal water cost of carbon gain (*∂E/∂A*)—is typically assumed to be constant over short timescales and adjusted to soil water availability over long timescales (Manzoni et al., 2013). Experimental evidence supporting this framework has been reported for numerous species (Buckley, 2005). However, findings in more complex environments are mixed, prompting further debates on its foundations and applicability. Katul et al. (2010) observed the individual leaf *λ* of *Pinus taeda* L. of North Carolina was steady on short-term but decreased with elevated atmospheric CO_2_ resulting from prescribed burning. The fluctuations in *λ* with temperature were also observed on *Oryza sativa* L. and *Triticum aestivum* L (Huang et al., 2021). Severe water or heat stress disrupt leaf internal hydraulic and biochemical status, imposing hydraulic failure or non-stomatal limits that violate the optimality assumption (Marchin et al., 2023; Potkay et al., 2025b).

Although uncertainties regarding *λ* variability and model implementation persist, the optimality theory continues to be widely adopted in both empirical and modeling studies (Wang et al., 2020). Finally, two approaches have emerged for applying the optimality theory (Knauer et al., 2018; Manzoni et al., 2011): 1) deriving *λ* analytically from plant gaseous exchange measurements and using its variation to diagnose plant stress; 2) predicting plant gaseous exchange using a prescribed *λ*. These two applications constitute the focus of our study, which applies the optimality theory to a citrus orchard in southwestern China and evaluates its applicability.

Directly solving the Lagrange multiplier under optimal hypothesis (*λ=∂E/∂A*) is inherently challenging (Buckley and Mott, 2013). Some researchers estimate instantaneous *λ* by combining foliar environment variables with gas exchange measurements through physically based or empirical formulations (Medlyn et al., 2013; Volpe et al., 2011). The accuracy of *λ* derived by these approaches depends critically on the quality of input data and the suitability of the computational method employed (Thomas et al., 1999). Beyond gas exchange-based methods, characteristic *λ* values can also be inferred from correlated factors such as vegetation type, soil moisture, plant water potential, and atmospheric CO_2_ concentration (Lin et al., 2015; Liu et al., 2022; Wang et al., 2019).

From a theoretical perspective, optimal stomatal conductance models provide a mechanistic framework linking carbon assimilation and water loss through the Lagrange multiplier λ, offering a physiologically interpretable basis for predicting gas exchange. By reversing the causal direction in the calculation and prescribing an empirically known *λ* first, gas exchange can be predicted from environmental factors (Mrad et al., 2019). Transforming *λ* into *g_s_* requires gas exchange equations, typically Fick’s law of gas diffusion and the Farquhar biochemical model due to their mechanistic basis and minimal empiricism (Medlyn et al., 2011). These coupled optimal stomatal conductance-based models (OSCMs) can simultaneously solve for stomatal conductance (*g_s_*), photosynthesis (*A*), transpiration (*E*) and intercellular CO_2_ concentration (*c_i_*) using species traits and atmospheric inputs without cumbersome calibration work. Within this framework, plant gas exchange variables are often predicted with high fidelity (Buckley and Mott, 2013). Ji et al. (2017) predicted the *g_s_* of well-watered soybean and maize with R^2^ value of 0.86 and 0.88, respectively. Lu et al. (2016) successfully captured the responses of *A*, *E* and water use efficiency (*WUE*) along rainfall gradients across diverse forest sites.

In addition, two uncertainties arise in the application of the OSCMs. First, as research on stomatal behavior advances, evidence suggest that using hydraulic structural risks rather than transpiration water loss may more accurately represent the water cost for plants (Dewar et al., 2018; Wolf et al., 2016). Accordingly, the assumption of a constant Lagrange multiplier *λ* should be applied with caution, especially in arid regions (Potkay et al., 2025a; Venturas et al., 2018). Second, within the sub-equation of the OSCMs, the Farquhar functions adopt two different forms depending on photosynthetic limitations: either the CO_2_ carboxylation rate (Rubisco) or the electron transport rate (RuBP) (Vico et al., 2013). Some studies have preferred to assume a single dominant limitation to achieve a neat analytical expression and smooth solutions (Katul et al., 2010; Schymanski et al., 2015). However, in natural environments, limitations may shift due to fluctuations in water availability, light, temperature, and various other abiotic factors (Abdulbaki et al., 2022; Perdomo et al., 2017).

In this study, two years of citrus leaf gas exchange data were collected from a citrus orchard in southwestern China. Analytical *λ* under diverse meteorological conditions were examined and the applicability of the conventional optimal stomatal model was discussed. Our objectives were to: 1) calculate the marginal water cost of carbon gain *λ* and determine its characteristic values for citrus; 2) modify the optimal stomatal conductance-based models (OSCMs) by distinguishing Rubisco and RuBP scenarios, acknowledging that photosynthetic limitations may vary under different environmental conditions, and predict citrus gas exchange; 3) analyze the main factors affecting model performance and recommend suitable environmental conditions for its application. This research aims to enhance understanding of citrus foliar gas exchange and stomatal regulation in seasonal arid regions and provide a low-input method for forecasting agricultural water-carbon fluxes.

## 2 Materials and methods

### 2.1 Experimental site

**Figure 1:**
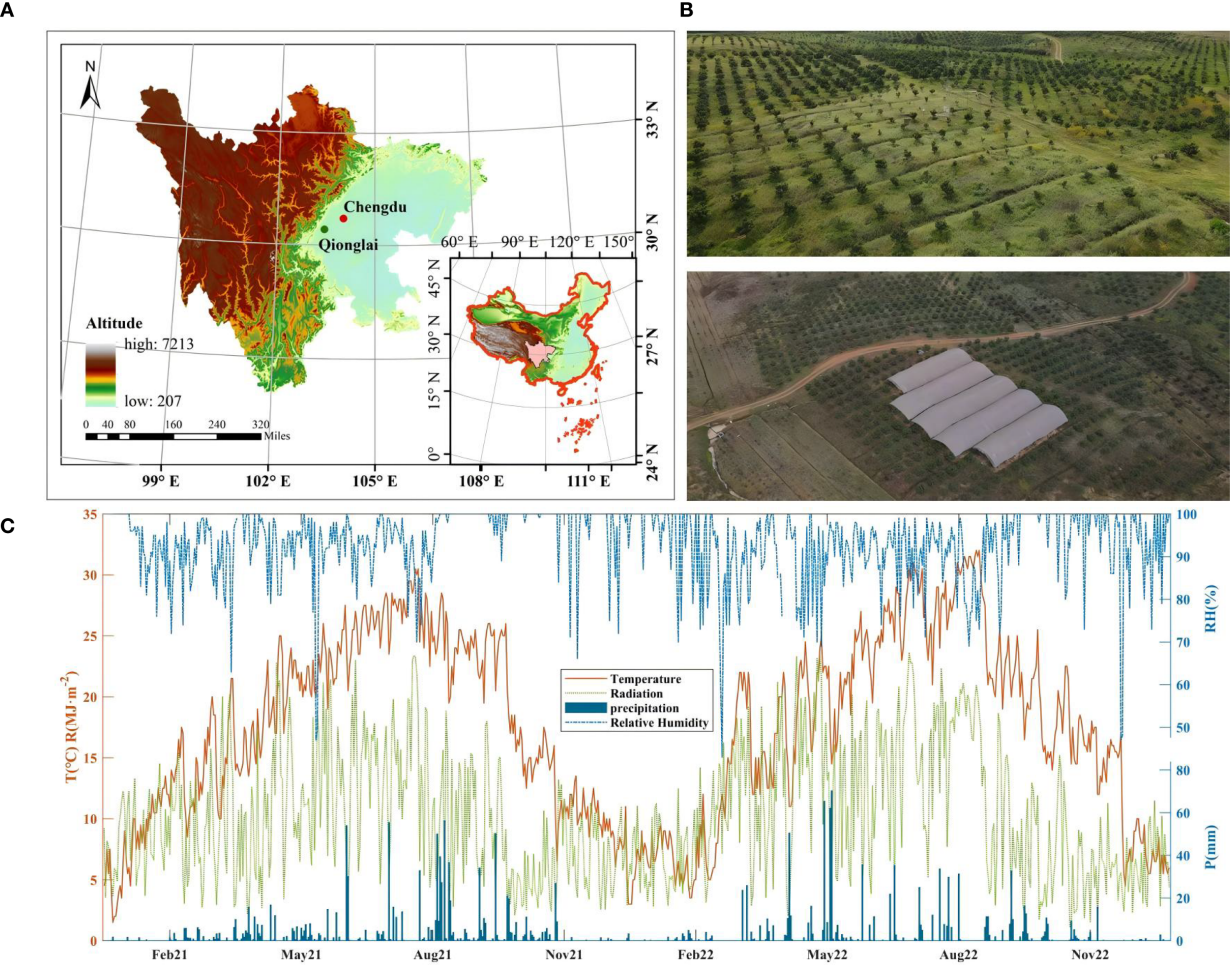
Geographic location and climatic condition of experimental site.

The measurements were conducted on six-year-old citrus (*Citrus Tachibana* Tanaka.) grafted onto tangerine rootstock (*Citrus Reticulata* Blanco.) during the phenological period (March–November) in an artificial citrus orchard in Qionglai city, Sichuan province, China (103.45°E, 30.34°N). The citrus orchard is situated at an altitude of 547 m in a subtropical monsoon climate zone characterized by abundant but seasonally uneven rainfall and solar radiation. The multi-year averages of air temperature, relative humidity, and annual precipitation were 16.8 °C, 82%, and 1041.3 mm, respectively. During the two experimental years, the mean air temperature, humidity, solar radiation, and annual precipitation were 17.6 °C, 93.2%, 10.2 MJ·m^-2^·day^-1^, and 1013.7 mm, respectively (**Figure 1**).

The experimental aerated greenhouse was located in a shallow hilly zone, with an average soil bulk density of 1.13 g·cm^-^³ and a maximum water-holding capacity (*θ_f_*) of 35.4% volume moisture content. Soil moisture was maintained at 60–75% *θ_f_* through irrigation, with total irrigation amounts of 347.56 and 313.48 mm in 2021 and 2022, respectively. Fertilization during the entire phenological period totaled 1167 kg·hm^-2^, with a nutrient composition of 19-19-19 (N-P_2_O_5_-K_2_O) percent by weight. According to conventional management, the proportions of basal fertilizer and topdressing applied during the shooting, fruit setting, and fruit expanding stages were 2:1:3:4. Citrus trees were planted at a spacing of 4 m × 3 m. Vigorous citrus trees of similar growth, with height of 2.3–2.5 m, were selected for observation.

### 2.2 Measurement

#### 2.2.1 Measurements of leaf gas exchange

**Figure 2:**
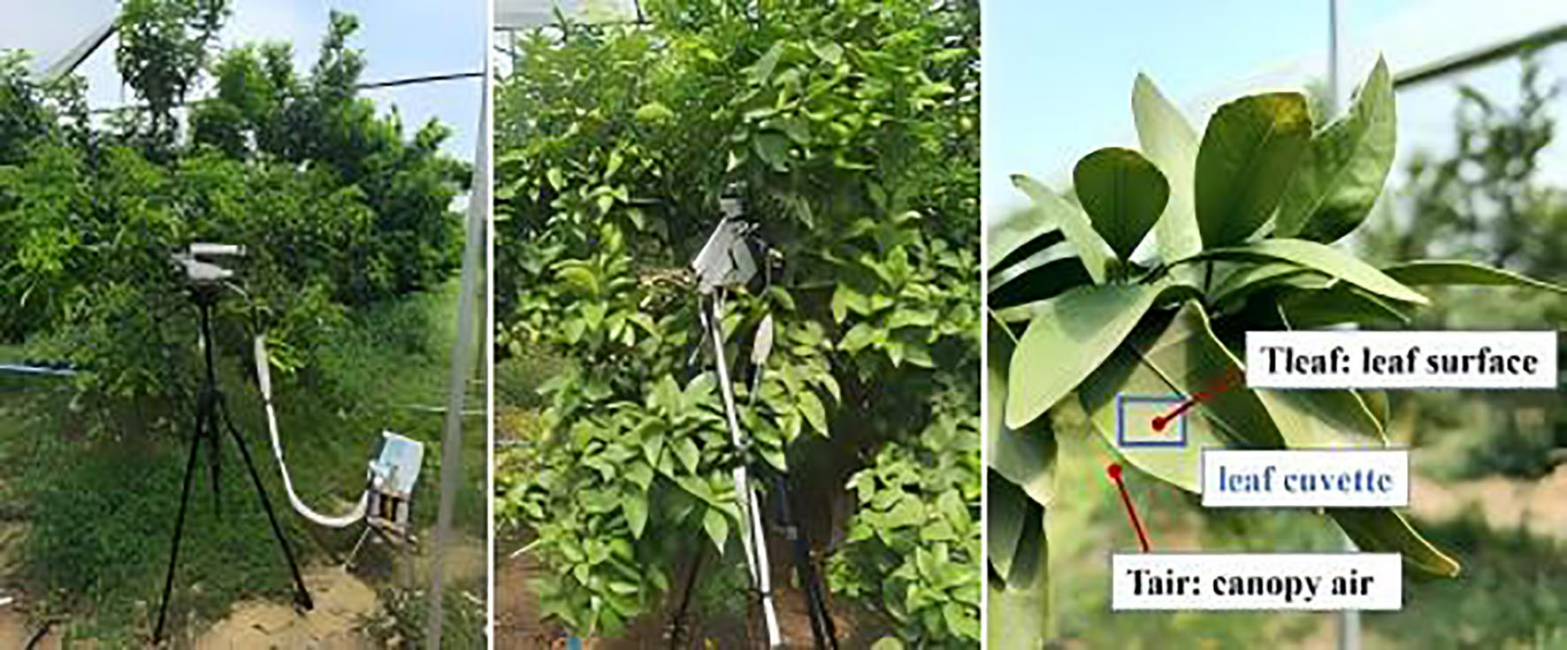
Leaf gas exchange measurements using a portable photosynthesis system.

Fully expanded citrus leaves on secondary shoots of similar sizes were selected for photosynthetic gas exchange measurements. For each tree, 4–6 leaves from two opposite orientations were tested without strictly distinguishing between sunlit and shaded positions (**Figure 2**). Gas exchange measurements were conducted as instantaneous observations on multiple leaves sampled at different times of the day; on full-day measurements, leaves measured in the morning were re-measured in the afternoon. Measurements were performed on rain-free days from April to October using infrared gas analysis systems (LI-6400XT, LI-COR Inc., Arizona, USA; Lcpro-SD, ADC Ltd., Hertfordshire, UK). At the start of each measurement day, the instrument was preheated for 30 minutes to routine check, including verifying the seal integrity of the leaf chamber and gas path, ensuring chemical effectiveness, and eliminating analyzer zero offset to guarantee data reliability. After validation, the selected functional leaves were tested *in situ* under ambient light conditions. A total of 1001 and 851 leaf gas exchange datasets were collected in 2021 and 2022, respectively (**Table 1**). 

**Table 1:** Summary of leaf gas exchange measurement dates and environmental conditions.

Order	Date (yyyy-mm-dd)	Time	Measure	Weather	Average soil moisture (%)
1	2021-04-29	9:00-17:00	325	sunny	26.49
2	2021-06-29	9:30-12:00	80	overcast	26.60
3	2021-07-02	10:00-18:00	141	cloudy	24.45
4	2021-07-03	10:00-18:00	115	overcast	22.66
5	2021-07-20	10:00-17:00	67	overcast	21.66
6	2021-07-31	9:00-17:30	113	sunny	26.75
7	2021-08-02	9:30-10:30	37	cloudy	26.06
8	2021-08-06	9:30-10:30	37	sunny	24.09
9	2021-09-15	9:00-12:00	86	cloudy	24.43
10	2022-04-29	9:00-12:00	26	cloudy	23.62
11	2022-06-24	9:00-12:00	28	cloudy	26.70
12	2022-07-06	9:00-16:00	302	overcast	–
13	2022-07-07	9:00-12:00	226	overcast	–
14	2022-07-21	9:00-12:00	86	overcast	26.80
15	2022-07-22	10:00-13:00	76	overcast	26.80
16	2022-08-09	9:00-11:30	26	cloudy	22.58
17	2022-10-01	10:00-10:30	81	cloudy	26.83
sum			1852		

#### 2.2.2 Measurements of leaf net photosynthesis-CO_2_ response (*A-c_i_*) curve

On 22^nd^ April and 20^th^ October 2022, the response of functional leaves to varying CO_2_ concentrations were measured using a LI-6400XT system equipped with RGB red-blue light sources and a CO_2_ injection device. Prior to measurements, an eight-point calibration was performed to minimize the influence of ambient air fluctuations. Leaves were first acclimated for 30 min at a photosynthetically active radiation of 1000 μmol·m^-2^·s^-1^ and a CO_2_ concentration of 400 μmol·mol^-1^ until gas exchange parameters stabilized. Subsequently, CO_2_ concentrations in the cuvette were adjusted stepwise, and measurements at each level were recorded after stabilization or after a maximum of 5 min. The CO_2_ sequence was set as follows: 400, 300, 200, 150, 100, 50, 400, 400, 600, 800, 1000, 1200, 1500, 1800, and 2000 μmol·CO_2_·mol^-1^. During the curve measurements, temperature and humidity were not actively controlled to conserve battery power. However, both variables remained relatively stable throughout each test, and the resulting uncertainties were considered acceptable.

### 2.3 Parameter acquisition and model development

#### 2.3.1 Calculation of photosynthetic characteristic parameters

The parameters including *K_m_*, Γ^*^, *V_cmax25_*, *J_max25_*, and *R_d25_* were fitted from *A-c_i_* curves based on the photosynthetic biochemical FvCB model and were assumed to be either constant or temperature-dependent, representing the photosynthetic traits of the studied citrus species. Under natural conditions, photosynthetic TPU limitation is rarely reached, rapidly transitioning to Rubisco-limited or RuBP-regeneration-limited states (Duursma, 2015; Rogers et al., 2021). Therefore, only the Rubisco and RuBP limitations were considered in this study. The FvCB model are shown in **Equations 1**–**3** (Farquhar et al., 1980; Sharkey, 1985):

(1) $$\text{A}=\text{min}\{{\text{w}}_{\text{c}},{\text{ w}}_{\text{J}}\}(1-\frac{{\text{Γ}}^{*}}{{\text{c}}_{\text{c}}})-{\text{R}}_{\text{d}}$$

(2) $${\text{w}}_{\text{c}}=\frac{{\text{V}}_{\text{cmax}}\cdot {\text{c}}_{\text{c}}}{{\text{c}}_{\text{c}}+{\text{K}}_{\text{m}}},\;{\text{K}}_{\text{m}}={\text{K}}_{\text{c}}(1+\frac{\text{O}}{{\text{K}}_{\text{o}}})$$

(3) $${\text{w}}_{\text{J}}=\frac{\text{J}\cdot {\text{c}}_{\text{c}}}{4{\text{c}}_{\text{c}}+8{\text{Γ}}^{*}},\;\text{J}=\frac{\text{α}\cdot \text{Q}}{{\left(1+\frac{{\text{α}}^{2}{\text{Q}}^{2}}{{\text{J}}_{\text{max}}^{2}}\right)}^{0.5}}$$

Where *A* is the net photosynthetic rate, μmol·m^-2^·s^-1^; *w_c_* and *w_J_* are the limited carboxylation rate by Rubisco and RuBP, respectively, μmol·m^-2^·s^-1^; Γ^*^ is the CO_2_ compensation concentration at chloroplast thylakoids, μmol·mol^-1^; *R_d_* is the rate of mitochondrial respiration under light, μmol·m^-2^·s^-1^; *V_cmax_* is the maximum rate of Rubisco carboxylation activity, μmol·m^-2^·s^-1^; *K_c_* and *K_o_* are the Michaelis Menten coefficients of Rubisco activity for CO_2_ and O_2_, respectively; *O* is the intercellular O_2_ concentration, mmol·mol^−1^; *J* is the rate of electron transport, μmol·m^-2^·s^-1^; *J_max_* is the potential maximum rate of electron transport, μmol·m^-2^·s^-1^; *Q* is the photosynthetic photon flux density, μmol·m^-2^·s^-1^; *α* is the quantum yield of electron transport, dimensionless; *c_c_* is the CO_2_ concentration in the carboxylation site of chloroplast thylakoids, μmol·mol^-1^.

Parameters *V_cmax_* and *J_max_* are temperature-dependent and can be estimated from *A-c_i_* curves, then normalized to 25°C using **Equation 4** (Medlyn et al., 2002):

(4) $$\text{f}({\text{T}}_{\text{k}})={\text{k}}_{25}\text{exp}[\frac{{\text{E}}_{\text{a}}({\text{T}}_{\text{k}}-298)}{\left(298{\text{RT}}_{\text{k}}\right)}]\frac{1+\text{exp}(\frac{298\Delta \text{S}-{\text{H}}_{\text{d}}}{298\text{R}})}{1+\text{exp}(\frac{{\text{T}}_{\text{k}}\Delta \text{S}-{\text{H}}_{\text{d}}}{{\text{T}}_{\text{k}}\text{R}})}$$

Where *T_k_* is leaf kelvin temperature, K; *R* is the gas constant, J·mol^-1^·K^-1^; *k_25_* is the biochemical parameters including *V_cmax25_* and *J_max25_*, μmol·m^-2^·s^-1^; *ΔS* is entropy term, J·mol^-1^·K^-1^; *E_a_* is the rate of exponential increase of the biochemical parameters below the optimum temperature, J·mol^-1^; *H_d_* is the rate of decrease of the biochemical parameters above the optimum temperature, J·mol^-1^. The detailed values of *ΔS*, *E_a_*, and *H_d_* for *V_cmax25_* and *J_max25_* are shown in **Table 2**.

**Table 2:** List of symbol descriptions, units, source and default parameter values used in model calculations.

Symbol	Descriptions	Units	Source	Value
*A*	net photosynthetic rate	μmol·m-2·s-1	meas	
*E*	transpiration rate	mmol·m-2·s-1	meas	
*g_s_*	stomatal conductance to water	mol·m-2·s-1	meas	
*c_i_*	intercellular CO2 concentration	μmol·mol-1	meas	
*Q*	photosynthetic photon flux density	μmol·m-2·s-1	meas	
*T_a_*	Leaf surface air temperature	°C	meas	
*T_l_*	leaf temperature	°C		*T_a_*
*T_k_*	leaf kelvin temperature	K		*T_l_* + 273.15
*D*	vapor pressure deficit	kPa	meas	
*c_a_*	ambient CO2 concentration	μmol·mol-1	meas	
*P*	air pressure of the atmosphere on leaf surface	kPa	meas	
*w_c_*	limited carboxylation rate by Rubisco	μmol·m-2·s-1	Eq. 2	
*w_j_*	limited carboxylation rate by RuBP	μmol·m-2·s-1	Eq. 3	
Γ^*^	CO2 compensation concentration at chloroplast thylakoids	μmol·mol-1	Eq. 5	
*R_d_*	mitochondrial respiration rate under light	μmol·m^-2^·s^-1^	Eq. 4	
*R_d25_*	mitochondrial respiration rate under light at 25°C	μmol·m^-2^·s^-1^	cali	1.77
*V_cmax_*	maximum rate of Rubisco carboxylation activity	μmol·m^-2^·s^-1^	Eq. 4	
*V_cmax25_*	maximum rate of Rubisco carboxylation activity at 25°C	μmol·m^-2^·s^-1^	cali	43.21
*K_m_*	Michaelis Menten coefficients of Rubisco activity	μmol·mol^-1^	Eq. 2	
*K_c_*	Michaelis Menten coefficients of Rubisco activity for CO2	μmol·mol^-1^	Eq. 6	
*K_o_*	Michaelis Menten coefficients of Rubisco activity for O2	mmol·mol-1	Eq. 7	
*O*	intercellular O2 concentration	mmol·mol-1	coef	210
*J_max_*	potential maximum rate of electron transport	μmol·m^-2^·s^-1^	Eq. 4	
*J_max25_*	potential maximum rate of electron transport at 25°C	μmol·m^-2^·s^-1^	cali	75.56
*J*	electron transport rate	μmol·m^-2^·s^-1^	Eq. 3	
*α*	quantum yield of electron transport		coef	0.24
*R*	gas constant	J·mol-1·K-1	coef	8.314
*ΔS*	entropy term	J·mol-1·K-1	coef	629.26 for *V_cmax_* 631.88 for *J_max_*
*E_a_*	rate of exponential increase of the biochemical parameters below the optimum temperature	J·mol-1	coef	58550 for *V_cmax_* 29680 for *J_max_*
*H_d_*	rate of decrease of the biochemical parameters above the optimum temperature	J·mol-1	coef	200,000
*g_m_*	mesophyll conductance	mol·m^-2^·s^-1^		infinity
*c_c_*	CO2 concentration in the carboxylation site of chloroplast thylakoids	μmol·mol^-1^		*c_i_*
*A_c_*	simulated net photosynthetic rate under Rubisco limitation	μmol·m-2·s-1	Eq. 9	
*A_j_*	simulated net photosynthetic rate under RuBP limitation	μmol·m-2·s-1	Eq. 9	
*λ*	marginal water cost for unit carbon assimilation	mol·mol-1	Eq. 13	

‘meas’ in source column indicates that the parameter was measured using a photosynthetic system; ‘cali’ indicates that the parameter was calibrated by *A-c_i_* curves; ‘coef’ indicates that the parameter was a preset coefficient; the ‘Eq.” indicates that the parameter was calculated using the corresponding equation.

Parameters Γ^*^, *K_c_*, and *K_o_* also depend on temperature, calculated by **Equations 5**–**7**:

(5) $${\text{Γ}}^{*}=42.75\text{exp}[\frac{37830({\text{T}}_{\text{k}}-298)}{\left(298{\text{RT}}_{\text{k}}\right)}]$$

(6) $${\text{K}}_{\text{c}}=274.6\text{exp}[\frac{80500({\text{T}}_{\text{k}}-298)}{\left(298{\text{RT}}_{\text{k}}\right)}]$$

(7) $${\text{K}}_{\text{o}}=419.8\text{exp}[\frac{14500({\text{T}}_{\text{k}}-298)}{\left(298{\text{RT}}_{\text{k}}\right)}]$$

Assuming the mesophyll conductance (*g_m_*) approaches infinity, the CO_2_ concentration at the carboxylation site (*c_c_*) can be approximated by the intercellular CO_2_ concentration (*c_i_*) (**Equation 8**):

(8) $${\text{g}}_{\text{m}}=\frac{\text{A}}{{\text{c}}_{\text{i}}-{\text{c}}_{\text{c}}},\;{\text{g}}_{\text{m}}\to \infty ,\;{\text{c}}_{\text{i}}={\text{c}}_{\text{c}}$$

**Figure 3:**
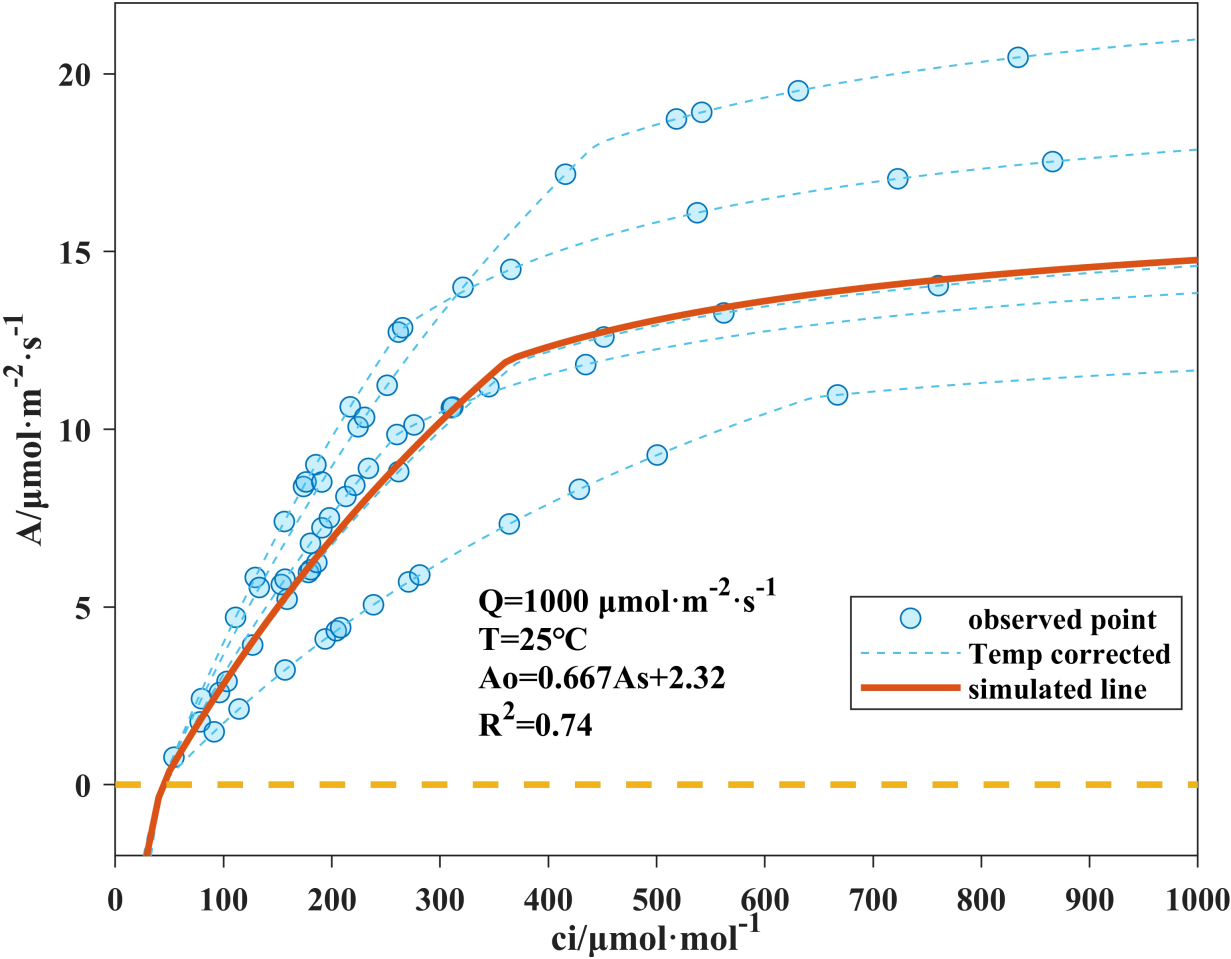
Fitted *A-ci* response curves. Points on the dashed lines are single-leaf measurements and corrected to 25 °C5 Solid lines are model simulations based on the average coefficients calibrated from the five measurement curves. *A_o_* denotes the temperature-corrected observation leaf photosynthesis rate; *A_s_* denotes the modeled rate calculated from the averaged coefficients at corresponding intercellular CO_2_ concentrations.

By substituting Equations 2, 3 into Equation 1, the simulated net photosynthetic rates under Rubisco limitation (*A_c_*) and RuBP limitation (*A_j_*) can be calculated, and the lower one was taken as the net photosynthesis rate. The photosynthetic characteristic parameters *K_m_*, Γ^*^, *V_cmax25_*, *J_max25_*, and *R_d25_* were fitted from *A-c_i_* response curve data using **Equation 9**, and the calibrated results are shown in **Figure 3**.

(9) $$\left\{ \begin{array}{c} {\text{A}}_{\text{c}}=\frac{{\text{V}}_{\text{cmax}}{\text{c}}_{\text{i}}}{{\text{c}}_{\text{i}}+{\text{K}}_{\text{m}}}(1-\frac{{\text{Γ}}^{*}}{{\text{c}}_{\text{i}}})-{\text{R}}_{\text{d}}\\ {\text{A}}_{\text{j}}=\frac{\text{αQ}({\text{c}}_{\text{i}}-{\text{Γ}}^{*})}{(4{\text{c}}_{\text{i}}+8{\text{Γ}}^{*}){\left(1+\frac{{\text{α}}^{2}{\text{Q}}^{2}}{{\text{J}}_{\text{max}}^{2}}\right)}^{0.5}}-{\text{R}}_{\text{d}} \end{array}\right.$$

#### 2.3.2 Calculation of the marginal water cost of carbon gain

The initial expression for the marginal water cost per unit carbon assimilation (*λ*) is given by **Equation 10**:

(10) $$\text{λ}=\frac{\partial \text{E}/\partial {\text{g}}_{\text{s}}}{\partial \text{A/}\partial {\text{g}}_{\text{s}}}\times 10^{3}$$

Where *A* is the net carbon assimilation rate, μmol·m^-2^·s^-1^; *E* is the water transpiration rate, mmol·m^-2^·s^-1^; *g_s_* is the stomatal conductance, mol·m^-2^·s^-1^; a factor of 10^3^ is applied for unit consistency.

Assuming that mesophyll and boundary conductances are large enough to have a negligible impact, *λ* can be calculated using **Equation 11** (Buckley et al., 2017):

(11) $$\text{λ=}\frac{\text{E}(\frac{1\mathrm{.6}}{{\text{g}}_{\text{s}}}+\frac{1}{\partial \text{A/}\partial {\text{C}}_{\text{i}}})}{\frac{1\mathrm{.6}}{{\text{g}}_{\text{s}}}\text{A}}$$

Based on the FvCB functions (Equation 9), *∂A/∂c_i_* can be obtained as the partial derivative of *A* with respect to *c_i_*. Consequently, *λ* can be calculated by **Equations 12**, **13**:

(12) $$\frac{\partial {\text{A}}_{\text{c}}}{\partial {\text{C}}_{\text{i}}}=\frac{{\text{V}}_{\text{cmax}}({\text{K}}_{\text{m}}+{\text{Γ}}^{*})}{{\left({\text{C}}_{\text{i}}+{\text{K}}_{\text{m}}\right)}^{2}},\;\frac{\partial {\text{A}}_{\text{j}}}{\partial {\text{C}}_{\text{i}}}=\frac{3\text{J}{\text{Γ}}^{*}}{4{\left({\text{C}}_{\text{i}}+2{\text{Γ}}^{*}\right)}^{2}}$$

(13) $$\left\{ \begin{array}{c} \text{λ}=\frac{\text{E}}{A}(1+\frac{{\text{g}}_{s}{\left({\text{c}}_{\text{i}}+{\text{K}}_{\text{m}}\right)}^{2}}{1.6{\text{V}}_{\text{cmax}}({\text{K}}_{\text{m}}+{\text{Γ}}^{*})})\;,{\text{ A}}_{\text{c}}<{\text{A}}_{\text{j}}\\ \text{λ}=\frac{\text{E}}{A}(1+\frac{{\text{g}}_{\text{s}}{\left({\text{c}}_{\text{i}}+2{\text{Γ}}^{*}\right)}^{2}{\left(1+\frac{{\text{α}}^{2}{\text{Q}}^{2}}{{\text{J}}_{\text{max}}^{2}}\right)}^{0.5}}{1.2\text{αQ}{\text{Γ}}^{*}})\;,{\text{ A}}_{\text{j}}<{\text{A}}_{\text{c}} \end{array}\right.$$

#### 2.3.3 Establishment of the *g_s_-E-A* coupled optimal stomatal conductance-based models

The coupled optimal stomatal conductance-based models (OSCMs) are constructed by integrating three components: Fick’s gas diffusion law (Equation 14), the FvCB biochemical model (Equation 9), and the constant-*λ* optimal stomatal regulation hypothesis (Equation 15). The OSCMs can solve *A*, *c_i_*, *E*, and *g_s_* simultaneously. The original equations are as follows:

(14) $$\left\{ \begin{array}{c} \text{E}={\text{g}}_{\text{s}}\frac{\text{D}}{\text{P}}\\ \text{A}=\frac{1}{1.6}{\text{g}}_{\text{s}}({\text{c}}_{\text{a}}-{\text{c}}_{\text{i}}) \end{array}\right.$$

(15) λ=constant

Where *P* is the atmospheric pressure at the leaf surface, kPa.

The final formulas derived for Rubisco limitation (OSCvc model) are given in **Equation 16**:

(16) $$\left\{ \begin{array}{c} \frac{\text{λP}}{1.6\text{D}}=\frac{1}{\left({\text{c}}_{\text{a}}-{\text{c}}_{\text{i}}\right)}+\frac{{\left(\frac{{\text{c}}_{\text{i}}+{\text{K}}_{\text{m}}}{{\text{c}}_{\text{a}}-{\text{c}}_{\text{i}}}\right)}^{2}(\frac{{\text{V}}_{\text{cmax}}({\text{c}}_{\text{i}}-{\text{Γ}}^{*})}{{\text{c}}_{\text{i}}+{\text{K}}_{\text{m}}}-{\text{R}}_{\text{d}})}{{\text{V}}_{\text{cmax}}({\text{K}}_{\text{m}}+{\text{Γ}}^{*})}\\ \text{A}=\frac{{\text{V}}_{\text{cmax}}({\text{c}}_{\text{i}}-{\text{Γ}}^{*})}{{\text{c}}_{\text{i}}+{\text{K}}_{\text{m}}}-{\text{R}}_{\text{d}}\\ {\text{g}}_{\text{s}}=\text{max}\{\frac{1.6}{\left({\text{c}}_{\text{a}}-{\text{c}}_{\text{i}}\right)}\text{A},{\text{ g}}_{\text{min}}\}\\ \text{E}={\text{g}}_{\text{s}}\frac{\text{D}}{P} \end{array}\right.$$

The final formulas derived for RuBP limitation (OSCvj model) are given in **Equation 17**:

(17) $$\left\{ \begin{array}{c} \frac{\text{λP}}{1.6\text{D}}=\frac{1}{\left({\text{c}}_{\text{a}}-{\text{c}}_{\text{i}}\right)}+\frac{1.6{\left(\frac{{\text{c}}_{\text{i}}+2{\text{Γ}}^{*}}{{\text{c}}_{\text{a}}-{\text{c}}_{\text{i}}}\right)}^{2}(\frac{\text{αQ}({\text{c}}_{\text{i}}-{\text{Γ}}^{*})}{4({\text{c}}_{\text{i}}+2{\text{Γ}}^{*})}-{\text{R}}_{\text{d}}{\left(1+\frac{{\text{α}}^{2}{\text{Q}}^{2}}{{\text{J}}_{\text{max}}^{2}}\right)}^{0.5})}{1{.2\alpha Q\Gamma }^{*}}\\ \text{A}=\frac{\text{αQ}({\text{c}}_{\text{i}}-{\text{Γ}}^{*})}{4({\text{c}}_{\text{i}}+2{\text{Γ}}^{*}){\left(1+\frac{{\text{α}}^{2}{\text{Q}}^{2}}{{\text{J}}_{\text{max}}^{2}}\right)}^{0.5}}-{\text{R}}_{\text{d}}\\ {\text{g}}_{\text{s}}=\text{max}\{\frac{1.6}{\left({\text{c}}_{\text{a}}-{\text{c}}_{\text{i}}\right)}\text{A},{\text{ g}}_{\text{min}}\}\\ \text{E}={\text{g}}_{\text{s}}\frac{\text{D}}{\text{P}} \end{array}\right.$$

The coupled optimal stomatal conductance-based models (OSCMs) are established as Equations 16, 17, corresponding to the OSCvc and OSCvj forms. In these models, once *c_i_* is determined from the topmost expression, it can be used in the subsequent equations to solve for *A*, *g_s_*, and *E*. To prevent negative simulated values of *g_s_*, a minimum stomata conductance (*g_min_*) of 0.01 mol·m^-2^·s^-1^ is imposed in this study.

The specific implementation steps of OSCMs are as follows: 1) Determining the photosynthesis characteristics parameters *V_cmax25_*, *J_max25_*, and *R_d25_* from *A-c_i_* curves and applying temperature correction; 2) Calculating the marginal water cost of carbon gain *λ* and determining a representative constant value; 3) Using OSCvc or OSCvj model to solve foliar gas exchange parameters including *c_i_*, *A*, *E*, and *g_s_.*

In steps 1–2, the characteristic coefficients are derived from surveyed gas exchange data, and can also be obtained from published studies on related species. In step 3, only *c_a_*, *P*, and *D*, are direct inputs for the OSCvc model (with *Q* added for the OSCvj model), while *T_l_* or *T_a_* is also necessary to adjust *K_m_*, Γ^*^, *V_cmax_*, *J_max_*, and *R_d_* to actual temperature. Finally, based on the OSCMs, foliar gas exchange (*A*, *E*, *c_i_*, *g_s_*) can be simulated using species-specific parameters (*V_cmax25_*, *J_max25_*, *R_d25_*, *λ*) and meteorological inputs (*c_a_*, *P*, *D*, *Q*, *T_a_*).

### 2.4 Analysis

#### 2.4.1 Analysis of parameter correlations

A structural equation model (SEM) incorporating meteorological parameters (*c_a_*, *D*, *Q*, *T_a_*), leaf gas exchange parameters (*A*, *E*, *c_i_*, *g_s_*), and the marginal water cost of carbon gain (*λ*) was established using SPSS Amos 28 (IBM Inc., Armonk, USA) to analyze the relationships between meteorological parameters and foliar gas exchange.

#### 2.4.2 Model evaluation

The performance of the OSCMs models is evaluated by four indicators (**Equations 18**–**21**). The determination coefficient (R^2^) indicates the goodness of model fit, while the mean absolute error (MAE), relative mean bias error (MBE) and absolute relative error (RE) reflect model accuracy in terms of magnitude and proportion.

(18) $${\text{R}}^{2}\;=\;\frac{(\sum_{\text{i}=1}^{\text{n}}({\text{O}}_{\text{i}}-\;\bar{\text{O}})\;{\text{(P}}_{\text{i}}-\bar{\text{P}}){)}^{2}}{\sum_{\text{i}=1}^{\text{n}}{\left({\text{O}}_{\text{i}}-\bar{\text{O}}\right)}^{2}\;\sum_{\text{i}=1}^{\text{n}}{\left({\text{P}}_{\text{i}}-\bar{\text{P}}\right)}^{2}},$$

(19) $$\text{MAE}=\frac{1}{\text{n}}\sum_{\text{i}=1}^{\text{n}}|{\text{P}}_{\text{i}}-{\text{O}}_{\text{i}}|$$

(20) $$\text{MBE}=\frac{\sum_{\text{i}=1}^{\text{n}}({\text{P}}_{\text{i}}-{\text{O}}_{\text{i}})}{\sum_{\text{i}=1}^{\text{n}}{\text{O}}_{\text{i}}}$$

(21) $$|\text{RE}{|}_{\text{i}}=\frac{\left|{\text{P}}_{\text{i}}-{\text{O}}_{\text{i}}\right|}{{\text{O}}_{\text{i}}}$$

Where P_i_ is the predicted value of gas exchange indicators, and O_i_ is the corresponding observed value; $\bar{\text{P}}$ and $\bar{\text{O}}$ are the mean values of predicted and observed datasets, respectively. Model performance improves as MAE, MBE, and RE decrease and R^2^ approaches 1.

#### 2.4.3 Model sensitivity

The model sensitivity coefficient quantifies the response of the model to variations in each input variable or parameter. A reference state was defined as an observed gas exchange data point under moderate meteorological conditions (*T_a_* = 35°C, *D* = 2.0 kPa). The sensitivity coefficient (SC) was then calculated from OSC model simulations with perturbed inputs using **Equations 22**, **23**:

(22) $${\text{SC}}_{\text{i}}=\frac{({\text{S}}_{\text{i}}-\text{S})/\text{S}}{({\text{F}}_{\text{i}}-\text{F})/\text{F}}$$

(23) $$\text{SC}=\frac{\sum_{\text{i}=1}^{\text{n}}{\text{SC}}_{\text{i}}}{\text{n}}$$

Where S is the observed gas exchange value at the reference state, including *g_s_*, *c_i_*, *A*, and *E*; S_i_ is the corresponding simulated value with adjusted inputs; F is the observed value of a model factor at the reference state, including *P*, *D*, *c_a_*, *Q*, *T_a_*, *λ*, *V_cmax25_*, *J_max25_*, and *R_d25_*; F_i_ is the corresponding adjusted value, with only one factor varied at a time; i is the adjustment level, i=1–8, corresponding to -20%, -15%, -10%, -5%, 5%, 10%, 15% and 20%; n is the number of adjustment levels, with n=8 in this study.

## 3 Results

### 3.1 Diurnal variability and representative values of *λ*

**Figure 4:**
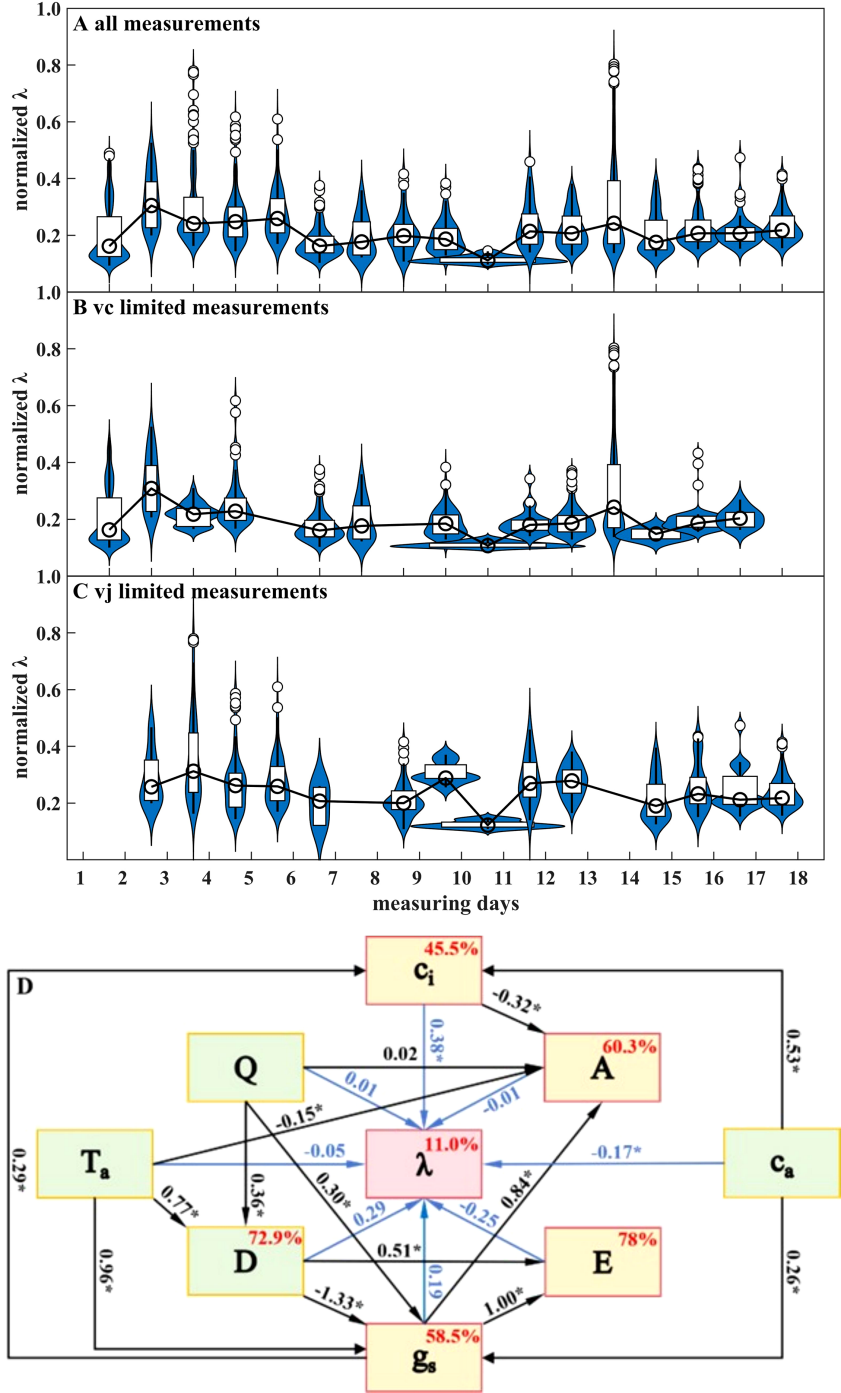
**(A–C)** Violin plots of normalized *λ* values across different measurement days under co-limited, *V_c_*-limited, and *V_j_*-limited conditions; **(D)** Standardized structural equation model (SEM) linking meteorological factors, leaf gas exchange variables, and *λ*. Values in boxes indicate the explained variance, and numbers along the arrows represent standardized path coefficients. One standard deviation change in a source variable results in a corresponding deviation in the target variable. Asterisks indicate that the regression weight is significant at the 0.001 level. Standardized path coefficients exceeding 1 reflect the tight coupling among vapor pressure deficit, stomatal conductance, and transpiration, rather than model misspecification.

The numerical solutions of the marginal water cost of carbon gain (*λ*) within the 10–90% range for each measurement day, are shown in **Figures 4A–C** for all points, *V_c_*-limited, and *V_j_*-limited points, respectively. Values of *λ* exhibited diurnal variability with an average coefficient of variance (CV) of 55.0%, and the fluctuation expanded to 74.6% and 65.7% under *V_c_*, and *V_j_* limitations, respectively. Despite this variability, the median *λ* values for individual testing days were relatively stable. For the co-limited dataset, median *λ* ranged in 520.89–3135.39. The distribution range shifted to 360.87–3134.98 under *V_c_* limitation and 594.94–3248.67 under *V_j_* limitation. Since the *λ* values across different days were distributed within comparable ranges, diurnal variability was not explicitly considered, and the median value was adopted as the citrus characteristic value for subsequent simulations. Accordingly, *λ* was set to 1787.10, 1478.51, and 2703.65 under co-limited, *V_c_*-limited, and *V_j_*-limited conditions, respectively, reflecting differences in leaf carbon-water trade-offs under contrasting photosynthesis limitations.

To further explore the drivers of *λ* variability, a structural equation model (SEM) was constructed based on ambient meteorological variables, leaf gas exchange variables, and *λ* (**Figure 4D**). The environmental component (green blocks) comprised photosynthetic photon flux density (*Q*), air temperature (*T_a_*), vapor pressure deficit (*D*), and ambient CO_2_ concentration (*c_a_*). Among these, *Q*, *T_a_*, and *c_a_* were determined as independent variables, whereas *D* was modeled as jointly driven by *Q* and *T_a_*. The leaf component (yellow blocks) included net carbon assimilation rate (*A*), transpiration rate (*E*), intercellular CO_2_ concentration (*c_i_*), and stomatal conductance (*g_s_*). *A* and *E* were assumed to affect gas exchange indirectly through stomatal regulation, rather than exerting direct effects on each other. As a connected bridge, *g_s_* was affected by all environmental variables and, in turn, influenced all leaf gaseous exchange parameters. Based on this SEM, the combined explanatory rate of all eight indicators for *λ* only reached 11%. Among all meteorological factors, only *c_a_* exerted significant influence on *λ*, with a direct effect of -0.17 and an indirect effect of 0.25 through *c_i_* and *g_s_*. Among all variables, *Q*, *Ta*, and *ci* showed positive effects on *λ*, whereas E had a negative effect, and the remaining variables contributed negligibly (total effects<0.01). Meteorological and gas exchange variables were not the intrinsic drivers for *λ* variability.

### 3.2 Performances of the OSCMs

**Figure 5:**
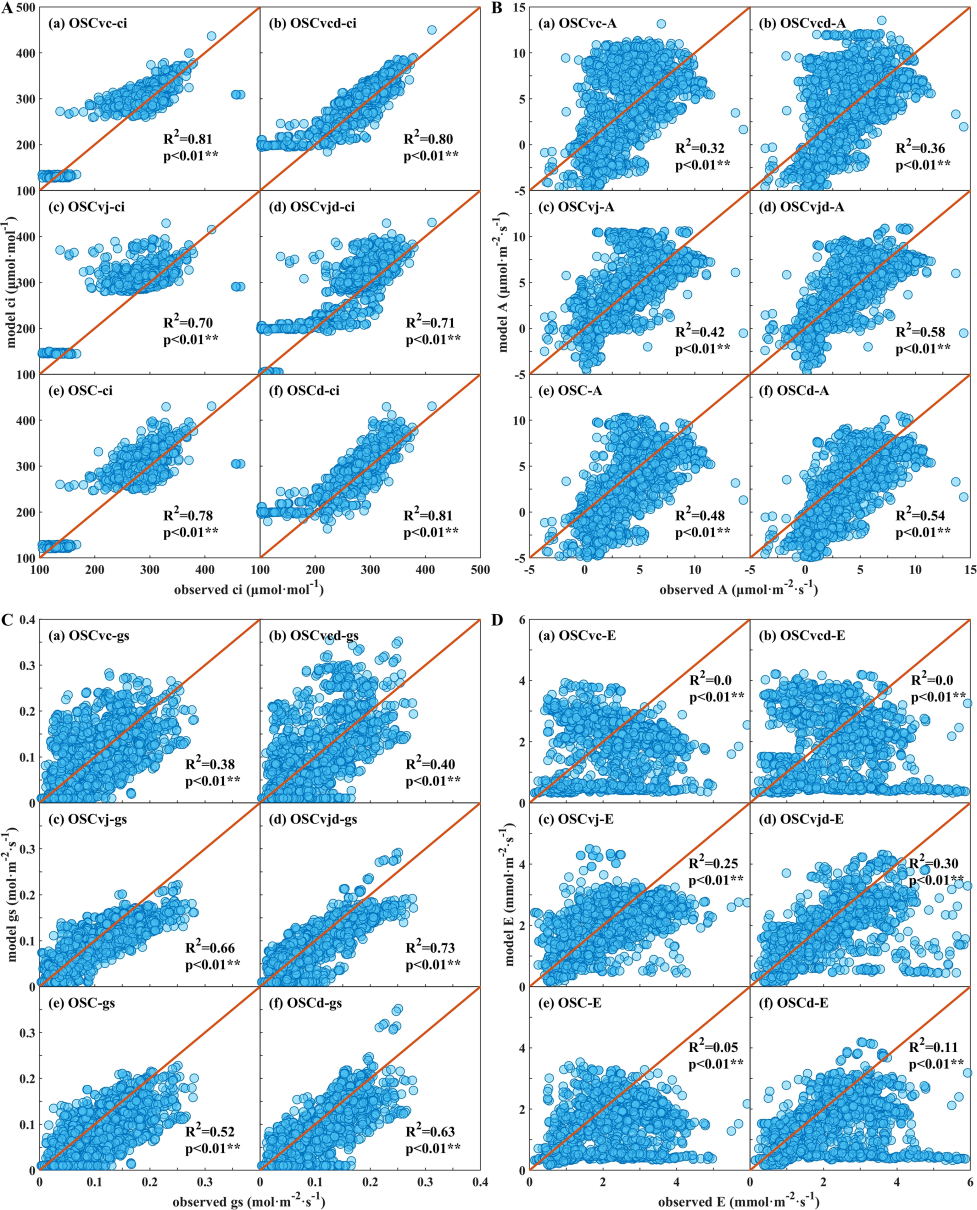
**(A)** Intercellular CO_2_ concentration (*c_i_*) simulated by OSCMs; **(B)** Net carbon assimilated rate (*A*) simulated by OSCMs; **(C)** Stomatal conductance (*g_s_*) simulated by OSCMs; **(D)** Transpiration rate (*E*) simulated by OSCMs. For the initial OSC, OSCvc, and OSCvj models, *λ* was set as the median value over the entire growth period, whereas for models with subscript “d”, *λ* represented the daily median for each testing day.

The simulation results of citrus leaf gas exchange parameters are presented in **Figure 5**. In the OSCvc and OSCvcd models, photosynthesis was assumed to be limited solely by Rubisco carboxylation rate and was calculated using Equation 16 (**Figures 5Ba, b**); in the OSCvj and OSCvjd models, photosynthesis was limited by RuBP recycle rate and calculated using Equation 17 (**Figures 5Bc, d**); in the OSC and OSCd models, photosynthetic limitations were distinguished by the method described in 2.3.1 (**Figures 5Be, f**). The specific *λ* values are shown in **Table 3**. The OSC, OSCvc, and OSCvj models employed the median *λ* values of the entire phenological period, whereas the OSCd, OSCvcd, and OSCvjd models used daily median *λ* values for each testing day.

**Table 3:** Diurnal representative values of λ across different measurement days.

Order	Date (yyyy-mm-dd)	Median value of *λ*	Median value of *λ* under vc limited	Median value of *λ* under vj limited
1	2021-04-29	520.89	525.86	–
2	2021-06-29	1,643.41	1,667.70	1,321.57
3	2021-07-02	2,138.93	1,780.99	3,248.67
4	2021-07-03	2,570.25	2,240.79	2,802.25
5	2021-07-20	2,922.37	–	2,922.37
6	2021-07-31	1,215.57	1,191.83	1,954.64
7	2021-08-02	1,458.09	1,458.09	–
8	2021-08-06	1,810.29	–	1,840.31
9	2021-09-15	1,625.71	1,599.26	3,208.17
10	2022-04-29	426.87	360.87	594.94
11	2022-06-24	2,029.29	1,511.00	2,888.72
12	2022-07-06	2,000.97	1,696.35	3,033.92
13	2022-07-07	3,135.39	3,134.98	–
14	2022-07-21	1,769.87	1,228.22	2,072.09
15	2022-07-22	2,443.56	2,032.39	2,958.37
16	2022-08-09	2,448.98	2,378.56	2,548.55
17	2022-10-01	2,689.75	–	2,691.51
median		1,787.10	1,478.51	2,703.65

For all four gas exchange parameters, models using daily *λ* outperformed those using long-term *λ*, highlighting the importance of the main parameter *λ*. For *c_i_* simulation, all models achieved high R^2^ of 0.70–0.81 (**Figure 5A**). For *A* and *g_s_*, the OSCvjd model performed best with R^2^ values of 0.58 and 0.73, respectively. Without daily *λ* inputs, the OSC model provided the best *A* estimation with an R^2^ of 0.48 (**Figure 5Be**). In contrast, *E* estimation showed much lower accuracy with a maximum R^2^ of only 0.30 achieved by the OSCvjd model (**Figure 5Dd**).

**Figure 6:**
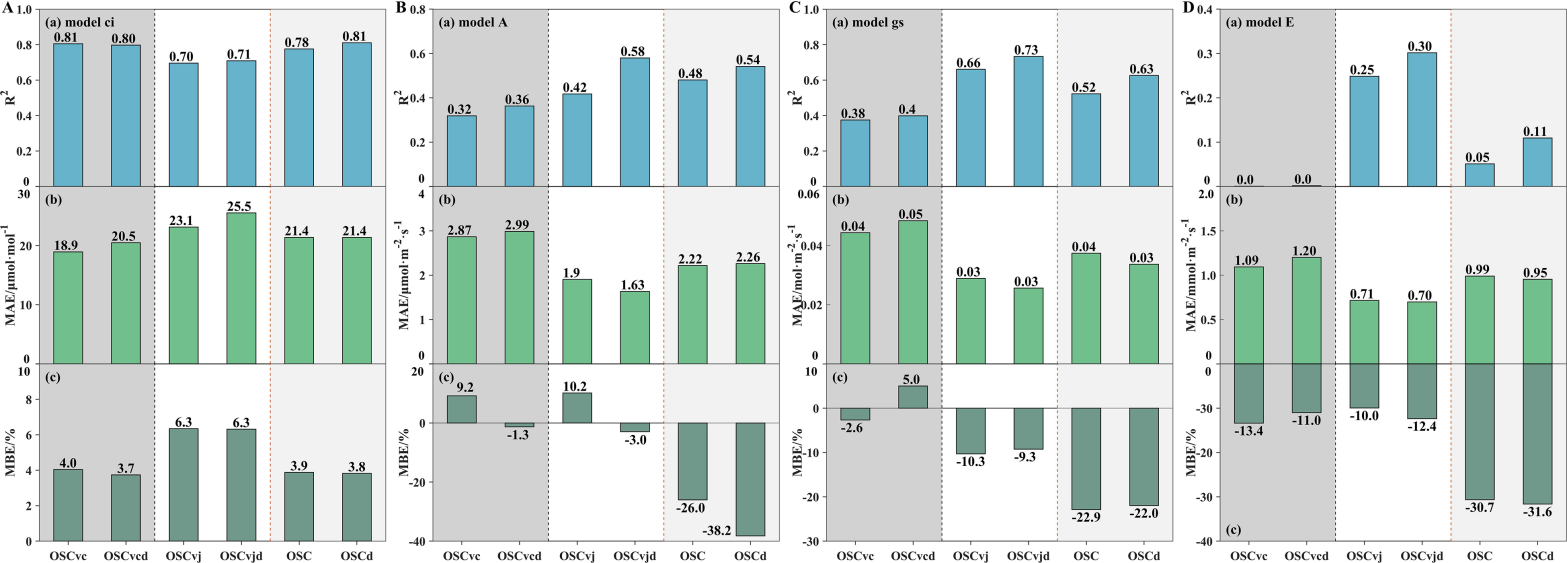
**(A)** Coefficient of determination (R^2^), mean average error (MAE), and relative mean bias error (MBE) of OSCMs for *c_i_* simulation; **(B)** Model performance for *A* simulation; **(C)** Model performance for *g_s_* simulation; **(D)** Model performance for *E* simulation.

The R^2^, MAE, and MBE values of OSCvc, OSCvcd, OSCvj, OSCvjd, OSC, and OSCd models are shown in **Figure 6**. In both solution schemes of the OSCMs formulations (Equations 16, 17), *c_i_* is calculated first and the OSCvc model got the highest accuracy with R^2^ of 0.81 and 0.80, MAE of 18.9 and 20.5 μmol·mol^-1^ under long-term and daily *λ* inputs, respectively. The OSCvj and OSC models also performed well with R^2^ of 0.70–0.81, MAE of 21.4–25.5 μmol·mol^-1^. All six OSCMs showed a slight overestimation tendency with MBE values of 3.7–6.3%.

In the second step of the coupled equations, *A* is calculated from *c_i_* using the Farquhar functions. Although the OSCvj model performed poorly in *c_i_* estimation, it obtained the highest R^2^ of 0.42–0.58 for *A* estimation without evident bias. The OSC model also showed acceptable R^2^ of 0.48–0.54 but exhibited a clear underestimation tendency with MBE values of -26.0–38.2%. In contrast, the OSCvc model showed the lowest accuracy with R^2^ of 0.32–0.36 and MAE of 2.87–2.99 μmol·m^-2^·s^-1^. These errors may stem from the inapplicability of the temperature correction coefficients (Equations 4–7), variability in photosynthetic characteristic parameters (**Table 4**), and the neglect of mesophyll resistance (Equation 8).

**Table 4:** The calibrated photosynthesis characteristics parameters derived from the FvCB model.

Order	T_l_	K_m_	Γ^*^	V_cmax25_	J_max25_	R_d25_
units	°C	μmol ·m^-2^·s^-1^	μmol ·mol^-1^	μmol ·m^-2^·s^-1^	μmol ·m^-2^·s^-1^	μmol ·m^-2^·s^-1^
1	31.06	1,173.59	54.60	39.09	68.87	1.45
2	33.13	1,406.20	60.43	56.58	86.11	2.58
3	29.18	1,000.11	50.21	24.53	54.20	1.72
4	36.69	1,911.34	71.66	43.98	65.02	2.30
5	27.81	885.32	46.47	51.85	103.61	0.81
average				43.21	75.56	1.77

In the final step, *g_s_* and *E* were calculated by *A* and *c_i_* based on Fick’s law of gas diffusion. For *g_s_* simulation, the OSCvjd model achieved the highest accuracy (R^2^ = 0.73, MBE=-9.3%). The OSCd model also showed good fit accuracy (R^2^ = 0.63) but exhibited clear underestimation (MBE = -22.0%). All OSCMs performed poorly in *E* simulation (R^2^ ≤ 0.30), showing pronounced underestimation trend with MBE of -10.0–31.6%. Estimation accuracy of all six OSCMs generally declined from *c_i_* to *A*, *g_s_*, and *E*, likely due to error propagation among the coupled gas exchange parameters.

Overall, the OSCvj and OSC methods exhibited acceptable performance in simulating citrus leaf *c_i_*, *A*, *g_s_*, and *E*. With long-term *λ* input, the OSC model outperformed the OSCvj model in *c_i_* and *A* estimation, whereas with daily *λ* input, the OSCvjd model performed best across all four gaseous exchange parameters. The superior performance of the OSC formulation for *A* supports the view that biochemical limitations shift over time, whereas the relatively better performance of the OSCvj formulation for *g_s_* and *E* suggests that light-limitation may be prevalent during our measurements.

### 3.3 Model error and sensitivity analysis of the OSC model

**Figure 7:**
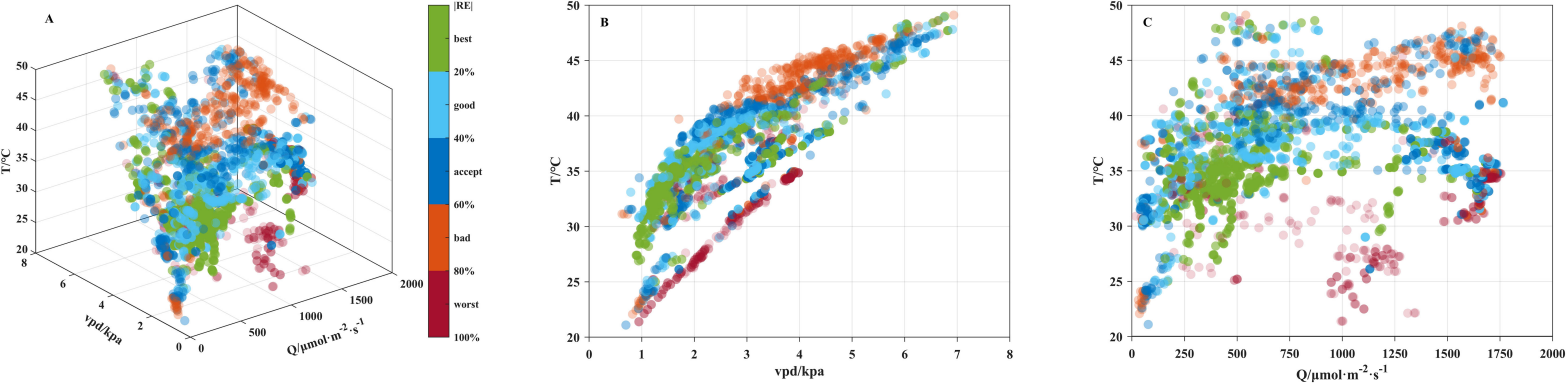
**(A)** Absolute relative error (|RE|) of *g_s_* simulated by the OSC model under varying *T*, *Q*, and *D* conditions; **(B)** |RE| of *g_s_* simulated the OSC model under varying *T* and *D* conditions; **(C)** |RE| of *g_s_* simulated the OSC model under varying *T* and *Q* conditions.

The OSC model was selected for further error analysis as it performed better with long-term *λ* input, which was easier to obtain. The absolute relative errors (|RE|) of *g_s_* simulated the OSC model under varying environmental *T_a_*, *Q*, and *D* conditions are shown in **Figure 7**. Data points with acceptable error (|RE |< 60%) were mainly distributed under moderated meteorological condition, characterized by relatively low *T_a_* and *D*. The best-performing estimates (green points, |RE|< 20%) were primarily found at *T_a_* of 30–40 °C and *D* of 1–2 kPa. Within this range, the mean |RE| and RE were 35.20% and -11.68%, respectively. When *T_a_* exceeded 40 °C or *D* was higher than 5 kPa, |RE| increased sharply reaching 71.17%. In contrast, no clear threshold was observed for *Q*.

**Figure 8:**
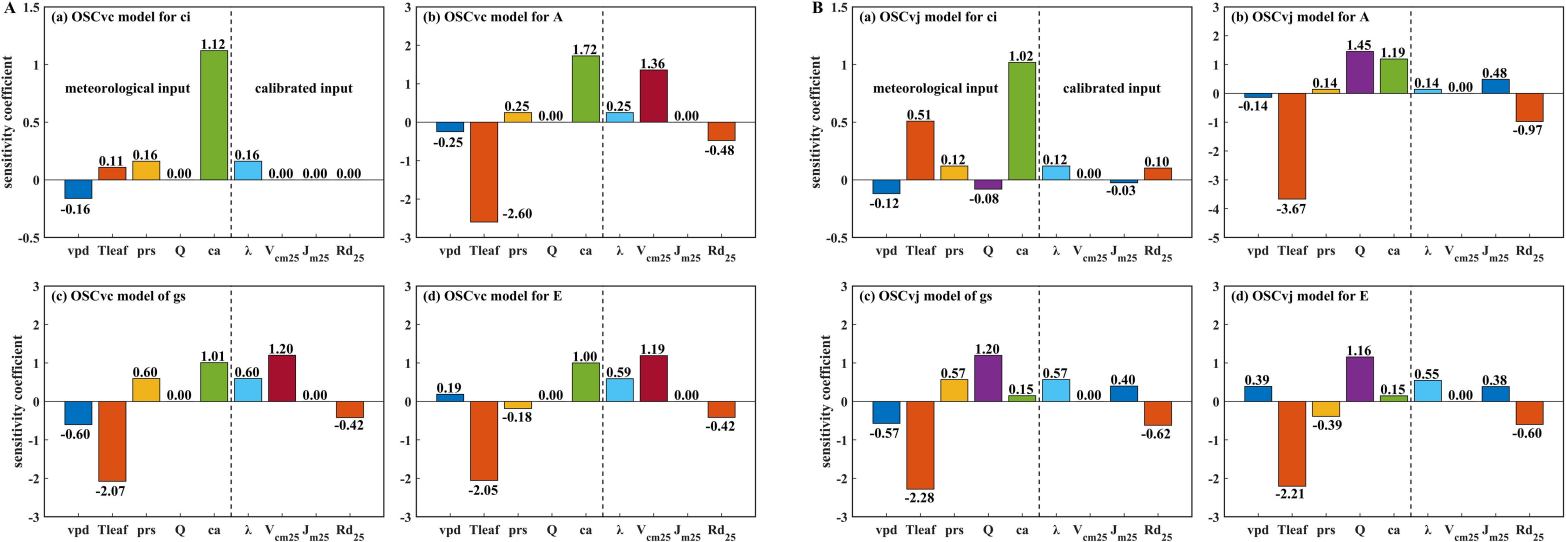
**(A)** Sensitivity coefficients of gas exchange variables (*c_i_*, *A*, *g_s_*, and *E*) simulated by *V_c_*-limited OSC model in response to meteorological inputs (*D*, *T_a_*, *P*, *Q*, and *c_a_*) and calibrated parameters (*λ*, *V_cmax25_*, *J_max25_*, and *R_d25_*); **(B)** Sensitivity coefficients of gas exchange variables simulated by *V_j_*-limited OSC model in response to model inputs.

The sensitivity coefficients (SC) of the four gas exchange parameters (*c_i_*, *A*, *g_s_*, and *E*) estimated by the *V_c_*-limited and *V_j_*-limited OSC models (Equations 16, 17) in response to meteorological inputs (*P*, *D*, *c_a_*, *Q*, and *T_a_*) and calibrated inputs (*λ*, *V_cmax25_*, *J_max25_*, and *R_d25_*) are shown in **Figure 8**. For *c_i_* simulation (**Figures 8Aa, Ba**), *c_a_* was the dominant factor in both OSC formulations, while photosynthesis capacity parameters (*V_cmax25_*, *J_max25_*, and *R_d25_*) were negligible effects. For the other three gas exchange parameters, *T_a_* was the most influential factor with the SCs ranging from -2.05 to -2.60 under *V_c_* limitation and from -2.21 to -3.67 under *V_j_* limitation. This may be because *T_a_* affects both leaf biochemical processes and stomatal regulation. In addition, the reference point was set near 35°C (Equation 22), a condition close to optimal for OSC simulation (**Figures 7B, C**), making deviations caused by *T_a_* fluctuations more pronounced.

Among the calibrated inputs, *V_cmax25_*, *J_max25_*, and *R_d25_* were used in the Farquhar biochemical model, while *λ* was defined by the optimal stomatal regulation theory. *V_cmax25_* had no effect on the *V_j_*-limited OSC model since the model (Equation 17) is independent of it. Similarly, *J_max25_* did not affect the *V_c_*-limited OSC model. The *V_c_*-limited OSC model was more sensitive to *V_cmax25_* (SC= -0.94) than to *R_d25_* (SC = -0.33), whereas the *V_j_*-limited OSC model was more sensitive to *R_d25_* (SC= -0.52) than to *J_max25_* (SC = 0.31). In addition to the photosynthesis capacity parameters, *λ* also influenced OSC model performance, while its sensitivity was smaller (average 0.40 and 0.34 under *V_c_* and *V_j_* limitation, respectively). Notably, the sensitivity of *g_s_* and *E* to *λ* (average 0.58 and 0.57) was higher than that of *c_i_* and *A* (average 0.14 and 0.19).

## 4 Discussion

### 4.1 Meteorological interactions and controls on gas exchange

The climate factors monitored in this study include leaf-surrounding atmospheric temperature, radiation, vapor pressure deficit, and CO_2_ concentration (*T_a_*, *Q*, *vpd*, *c_a_*). Among those meteorological factors, only *c_a_* showed significant effect on *λ* (**Figure 4d**). However, *c_a_* varies little over short periods and is often treated as a background value, so its influence on stomatal regulation is typically neglected. Over broader spatial and temporal scales, its impact would likely be more pronounced, particularly in the context of ongoing greenhouse (Frank et al., 2015; Kirschbaum, 2004).

For the other three factors, *D* is primarily controlled by *T_a_* and *Q* with an explanation rate of 72.9% (**Figure 4d**). In this study, measurements were conducted only on rainless days to avoid equipment damage. Under such conditions, atmospheric water vapor mainly originates from latent heat evaporation driven by radiation and temperature. By definition, *D* is the ratio of the atmospheric water vapor concentration to the saturation water vapor concentration at a given air temperature, and is therefore largely determined by *T_a_* and *Q* on rainless days.

The thermocouple measuring *T_a_* is positioned very close to the leaf surface, so *T_a_* is influenced by both solar heating and cooling from leaf transpiration. As shown in **Figure 7c**, *T_a_* initially increases with *Q*, but rises little once *T_a_* reaches around 40 °C. Thus, *T_a_* and *Q* are the two most dominant and relatively independent meteorological factors affecting foliar gas exchange.

In addition to heating the underlying surface, solar radiation directly supplies energy for plant photosynthesis. Fluctuations in natural light intensity are common (Vialet et al., 2017a), and photosynthetic limitations can shift even over short timescales (**Figures 4b, c**). Under low irradiance, insufficient photic capture limits the electron transport rate (*V_j_*), thereby restricting photosynthesis. As *Q* increases, sufficient photons drive light reactions, and photosynthesis becomes limited by the availability of reaction substrate, 3-phosphoglycerate (PGA) produced via RuBP carboxylation. Consequently, under high irradiance, the Rubisco carboxylation capacity (*V_c_*) becomes the primary limitation to photosynthesis (Liu and van Iersel, 2021; Ye et al., 2021). These shifts highlight the necessity of incorporating dynamic environmental conditions into gas exchange modeling.

### 4.2 Explanations of *λ* variance

The fundamental principle of optimal stomatal regulation theory is the trade-off between the benefits and costs of stomatal opening. The steady-*λ* hypothesis adopted in this study focuses on the foliar water-carbon exchange, defining transpired water loss as the cost and photosynthetic carbon assimilation as the benefit (Cowan and Farquhar, 1977). Although most calculated *λ* points fall within the range of thousands and cluster around the median value with small fluctuations, a few value points rise sharply even exceeding 10,000 (**Figure 4**). Several possible explanations for this phenomenon are proposed as follows: 1) The *λ* calculated using Equation 13 would change abruptly when photosynthetic limitation shift between *V_c_* and *V_j_*. Plant physiological parameters that may affect *λ*, such as hydraulic conductivity, were not considered (Eller et al., 2020; Manzoni et al., 2011; Sperry et al., 2016). 2) The estimates of *λ* may be biased because mesophyll resistance was negligible, an assumption that is often unrealistic (Flexas et al., 2008; Wistuba et al., 2008). In addition, photosynthetic capacities can vary dynamically (Dewar et al., 2018), and parameters calibrated from limited *A-c_i_* curves without strict environmental control may not be universally applicable. 3) If the ability to maintain a relatively stable *λ* is considered an indicator of stomatal regulation capacity, this capacity may be compromised under extreme environmental stress, leading to abrupt increase in *λ* (Mäkelä et al., 2002).

Similar variability in *λ* have also been observed under large shifts in atmospheric CO_2_, temperature, and vapor pressure deficit (Huang et al., 2021; Katul et al., 2010; Thomas et al., 1999). Fundamentally, these explanations are consistent in that water use in optimality theory should not only be represented solely by instantaneous leaf transpiration but also account for long-term hydraulic safety and whole-plant development.

The discrepancy between realistic *λ* and the constant *λ* used in the OSCMs highlights their inherent limitations. Especially at high *T_a_* and *D* conditions, large deviations may occur even under sufficient irrigation (60–75% *θ_f_*). To improve the accuracy of gas exchange simulations, additional constraints such as plant hydraulic safety, leaf water potential, and legacy effects should also be incorporated into the optimization framework (Dewar et al., 2018; Eller et al., 2020; Feng et al., 2022). Conversely, detecting such *λ* fluctuations may provide useful diagnostic information on plant status from gas exchange measurements. Overall, elucidating stomatal structural evolution, regulatory capacity, and optimization strategies under complex natural conditions remains a long-term challenge (Mäkelä et al., 2002). Further investigation will advance understanding of plant growth and stress resistance across species.

### 4.3 Application of the OSCMs

Errors in the OSCMs mainly originate from mismatches between prescribed parameters and their true physiological values (e.g., *V_cmax_*, *J_max_*, *K_m_*, Γ^*^, *R_d_*, *g_m_*, and *λ*). Among these parameters, *λ* is particularly problematic. Its calculation (Equation 13) is derived solely from gas exchange relationships without explicitly accounting for water availability. Consequently, the computed values should be interpreted as expected *λ* values during stomatal regulation, rather than the true shadow price of water. For example, *λ* estimated under *V_j_* limitation was generally higher than that under *V_c_* limitation (**Table 3**). Under *V_c_* limitation, *A* is primarily constrained by *c_i_*. Stomatal opening can therefore provide substantial carbon gain relative to water loss, leading the optimization equation to compute a low *λ* (a low apparent cost of stomata opening). In reality, *V_c_* limitation often arise from stomatal closure under water deficit, where the true *λ* should be high because water is more expensive. The inconsistency between expected and actual *λ* helps explain the poor performance of the OSCvc and OSCvcd models. In contrast, under *V_j_* limitation, *A* is primarily *Q*-limited because favorable plant water status allows stomata to remain open and maintain sufficient *c_i_*. Accordingly, the expected *λ* more closely reflects the real trade-off between stomatal opening benefit and water loss, resulting in better performance of the OSCvj and OSCvjd models.

Additionally, as discussed in 4.2, *λ* was assumed to be constant, whereas in reality it varies over time. Therefore, OSCMs using daily *λ* inputs generally performed better than those using long-term *λ* inputs (**Figures 5**, **6**). Moderate environmental conditions without soil or atmospheric drought are therefore recommended, as actual *λ* values are more likely to match the prescribed inputs. As shown in **Figure 7c**, errors in *g_s_* estimation by the OSC model increased rapidly when *T_a_* exceeded 40°C, indicating a breakdown of the optimal regulation assumptions and model validity. In this study, the OSC model performed best under conditions where *T_a_* and *D* ranged from 30–40°C and 1–2 kPa, respectively.

Although simplifying trait parameters as constants inevitably introduces errors, this approach remains feasible under limited input conditions. The required observational inputs are even fewer than those of single *g_s_* or *E* simulation models (Wang et al., 2013). Incorporating additional constraints into the optimization framework or adopting short-term analytic solutions for *λ* would improve its estimation accuracy and overall model performance. Further development of the OSCMs may proceed along two main directions. First, deeper mechanistic understanding and mathematical decomposition of optimal stomatal regulation are needed. Such approaches inevitably demands higher data requirements including root zone moisture, xylem vulnerability, mesophyll conductance, plant hydraulic flow, leaf water potential, and carbon allocation patterns (Dewar et al., 2018; Liang et al., 2018; Mencuccini et al., 2019; Wolf et al., 2016). Second, accounting for temporal lags in stomatal responses and explicitly incorporating them into model applications is essential, particularly under highly fluctuating natural environmental conditions (Holtzman et al., 2024; Vialet et al., 2017b). Beyond further refinement of the OSCMs, addressing the limitations of currently available field-scale meteorological observations calls for complementary strategies. Further research on the feedback of vegetation transpiration on near-surface temperature would simplify plant-scale observations and improve simulations of surface vegetation gas exchange under reduced input requirements. Constructing iterative algorithms based on the energy balance among incoming radiation, latent heat, and sensible heat provides an effective mechanistic approach (Wu et al., 2019).

## 5 Conclusions

This study applied optimal stomatal regulation theory with a constant marginal water-carbon conversion assumption on an artificial citrus orchard in a seasonal arid region of southwestern China. Photosynthetic limitations varied with environmental conditions, and characteristic *λ* values were identified as 1787.10, 1478.51, and 2703.65 for co-limited, *V_c_*-limited, and *V_j_*-limited conditions, respectively. The OSC-based models (OSCMs) achieved acceptable simulation performance across gas exchange variables, with the OSCvc model providing the highest accuracy for ci (R² = 0.81), the OSCvj model performing best for *g_s_* and *E* (R^2^ = 0.66 and 0.25, respectively), and the co-limited OSC model yielding the highest accuracy for *A* (R² = 0.48). Among these formulations, the OSC model showed comparatively stable performance across multiple variables, highlighting its robustness for integrated gas exchange simulations. This work applies optimal stomatal regulation in gas exchange simulation and offers implications for irrigation management and climate resilience in subtropical orchards.

## Data Availability

The original contributions presented in the study are included in the article/supplementary material. Further inquiries can be directed to the corresponding authors.
